# Expression of Chicken DEC205 Reflects the Unique Structure and Function of the Avian Immune System

**DOI:** 10.1371/journal.pone.0051799

**Published:** 2013-01-09

**Authors:** Karen Staines, John R. Young, Colin Butter

**Affiliations:** Avian Viral Diseases Programme, The Pirbright Institute, Compton Laboratory, Newbury, Berkshire, United Kingdom; University of Birmingham, United Kingdom

## Abstract

The generation of appropriate adaptive immune responses relies critically on dendritic cells, about which relatively little is known in chickens, a vital livestock species, in comparison with man and mouse. We cloned and sequenced chicken DEC205 cDNA and used this knowledge to produce quantitative PCR assays and monoclonal antibodies to study expression of DEC205 as well as CD83. The gene structure of DEC205 was identical to those of other species. Transcripts of both genes were found at higher levels in lymphoid tissues and the expression of DEC205 in normal birds had a characteristic distribution in the primary lymphoid organs. In spleen, DEC205 was seen on cells ideally located to trap antigen. In thymus it was found on cells thought to participate in the education of T cells, and in the bursa on cells that may be involved in presentation of antigen to B cells and regulation of B cell migration. The expression of DEC205 on cells other than antigen presenting cells (APC) is also described. Isolated splenocytes strongly expressing DEC205 but not the KUL01 antigen have morphology similar to mammalian dendritic cells and the distinct expression of DEC205 within the avian-specific Bursa of Fabricius alludes to a unique function in this organ of B cell diversification.

## Introduction

Specialized APC sample the environment and ensure that appropriate antigen-specific adaptive immune responses protect an animal from danger, by activation and expansion of specific effectors from a randomly generated anticipatory repertoire, a function common to all jawed vertebrates. The converse generation of anergy by tolerance or clonal deletion is essential to prevent the recognition of self and is also driven by antigen presentation.

The generation of de novo adaptive responses, including responses to vaccines, is primarily elicited by dendritic cells (DC), specialist leucocytes adapted for antigen capture, processing and presentation to T lymphocytes [1]. Knowledge of these cells in a target species is therefore crucial in finding the most effective means of vaccination. Chickens are the largest source of animal protein worldwide, and their protection from numerous pathogens is mostly achieved by vaccination. However our understanding of DC in this species is limited (reviewed in [2] [3]). The generation of bone-marrow derived DC (BM-DC) has recently been described [4] and the cloning of a number of DC-related genes has facilitated the transcriptional analysis of BM-DC responses [3], [5], [6]. Although chickens have a different repertoire of immune receptor molecules, with cytokines, chemokines and TLR differing from mammalian counterparts, in vitro responses of chicken BM-DC essentially reflect those described in biomedical species. Notwithstanding these observations, few tools have been developed for the study of chicken DC *in vivo*, the sole report being of the generation of antibodies to CD83 [7] in which the authors reported a unique phenotype and distribution of a CD83 expressing cell.

That avian lymphoid tissues differ structurally from those of mammals has been well documented (reviewed in [8]). In common with most non-aquatic birds chickens lack lymph nodes, the primary site in mammals of DC-T cell interaction, though they possess less organised lymphoid nodules [9] and lymphoid aggregates in other tissues. The avian spleen differs from that of mammals [10] in both architecture and function [11]. Monoclonal antibodies have been used to define non-lymphoid cells in the chicken spleen, though lack of molecular characterization of the binding specificities of these reagents limits interpretation [12].

The Bursa of Fabricius is the primary site of B cell development [13] where B cell diversity is generated in avian species (reviewed by Ratcliffe [14]). A pleated epithelium is packed with underlying follicles, full of developing B cells, divided into cortical and medullary regions by a separating layer of other cells. Dispersed amongst the B cells is a minor population of bursal secretory dendritic cells (BSDC), first described by Olah [15].

While the thymus of chickens is remarkable in possessing 14 lobes, its micro-architecture and function appear to be grossly similar to that of mammals. Education of T cells by self-recognition is presumed to take place as in mammals [16] and defects in this process produce autoimmunity [17].

DEC205 is a C-type lectin endocytic receptor of the mannose receptor family, first cloned in mouse [18] and named because of its molecular weight and principal distribution in mice on DC and Thymic epithelial cells. It is known to enhance antigen presentation via MHCII^+ve^ lysosomal compartments [19], to be involved in the cross-presentation of antigen [20] and the recognition of apoptotic and necrotic self [21]. We surmised that the cloning of chicken DEC205, and production of antibodies to study it's expression, would afford a valuable tool to further the understanding of antigen presentation by cells in the uniquely adapted immune organs of chickens. We also generated CD83 recognising antibodies, adding to the repertoire of available DC reagents, and allowing a comparison of the expression of this reported marker of mature (antigen presenting) DC [22] with the expression of DEC205, a molecule involved in antigen sampling.

Investigations with the DEC205 antibodies allowed us to study the expression of the molecule within bursa, spleen and thymus and on isolated splenocytes, including those enriched for adherent cells.

## Results

### Sequence of the chicken DEC205 gene

Human and mouse DEC205 peptide sequences were used to search all available chicken EST databases. Three contigs were constructed, with sequence similarity spanning 83% of the human gene and including the 5′ end of the coding sequence. PCR using primers based on these sequences (Figure S1 and Table S2) was used to amplify the whole cDNA sequence, from Line 0 spleen cDNA and Light Sussex bursa cDNA libraries, including the sequences in the gaps between contigs. Several strategies for cloning the predicted 5′ signal-peptide exon, present in a single EST, from cDNA libraries were unsuccessful. To confirm the contiguity of the 5′ exon, sequence was obtained from a chicken genomic BAC clone by primer walking from the 5′ end of the sequence successfully amplified by PCR (Figure S2 and Table S2). This yielded the expected exon sequence after crossing an intron with canonical splice sites.

The full length coding sequence determined from cDNA and BAC sequences was submitted to the EMBL database (Accession number AJ574899). This provided the primary evidence for the gene annotation in Ensembl (ENSGALG00000011153) and in REFSEQ (NM_001037836). The ENSEMBL annotation includes an additional transcript (ENSGALT00000038329) which has a single codon insertion at the splice acceptor site preceding exon 17 and a three codon insertion at the beginning of exon 30. The former results from use of the first instead of the second ag in the sequence agcagGTAGCA at the splice acceptor site. The latter uses a non-canonical splice acceptor. Since there is no cDNA evidence for either, this variant transcript is probably an artifact of the ENSEMBL pipeline and is unlikely to exist.

**Figure 1:**
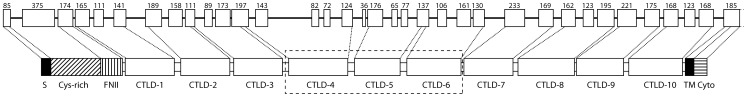
Structure of the chicken DEC205 gene. At the top, those parts of the 35 exons containing coding sequence are drawn to scale and labelled with their lengths in nucleotide residues. Introns are not to scale although their lengths relative to each other are approximately as drawn. The protein domain structure is shown below using shaded rectangles drawn to scale, with annotations: S, signal sequence; FNII, fibronectin type II; CTLD, C-type lectin like domain; TM, transmembrane region; Cyto, cytoplasmic region. Dotted lines connect the ends of domains to the encoding positions in exons. The dashed box indicates the three domains which were used to make the recombinant fusion protein used to raise monoclonal antibodies.

The exon structure of the chicken gene is shown in Figure 1, with its relationship to the conserved protein domains. It is essentially identical to the human DEC205 gene structure, with identical intron phases and only small differences in exon lengths. The chicken introns are shorter than the human equivalents, as is the case for most chicken genes. In the mouse, the one long transcript, ENSMUST00000028362, for which there is effective supporting evidence also has the identical gene structure.

Examination of the gene in its genomic context revealed a large upstream region with the order of many orthologous genes conserved in the human and chicken genomes, at least 1.6 million nucleotides in the chicken and 4 million in the human. This region of conserved gene order confirms that this is the chicken orthologue of the human LY75 (DEC205, CD205) gene. There is a break in conservation of gene order downstream of the chicken gene.

### Transcription of DEC205 and CD83 in tissues

**Figure 2:**
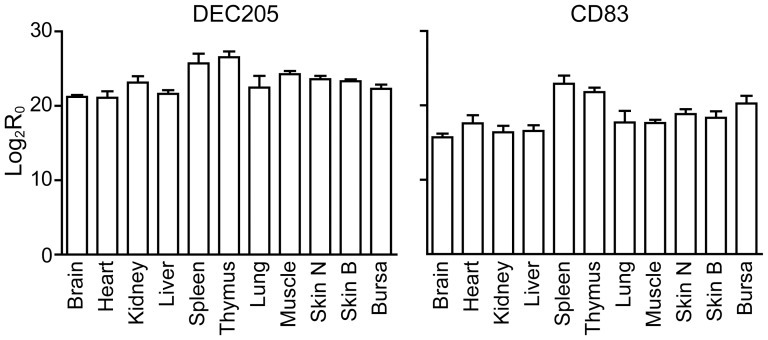
Transcript levels of DEC205 and CD83 genes in tissues. The vertical scale represents transcript levels relative to the reference 28S RNA on a logarithmic (base 2) scale. Bars indicate the levels in each of the tissues indicated below the horizontal axis. Error bars indicate the standard error of three assays carried out on RNA preparations from tissue samples from different birds.

Quantitative PCR measurements of transcript levels of DEC205 and CD83 genes are shown in Figure 2. Whilst there was constitutive expression in a range of tissues, there were significant differences in the level of either transcript (Anova; DEC205, P<0.002; CD83, P<0.0002). The transcript level of either gene in spleen and thymus was significantly higher than in all other tissues (Spleen: DEC205, P<0.01 for all tissues except bursa, skin and muscle; CD83, P<0.01 for all tissues except bursa. Thymus: DEC205, P<0.01 for all tissues; CD83, P<0.01 for all tissues except bursa).

### Monoclonal antibodies recognising chicken DEC205 and CD83

Taylor *et al.* [23], [24] showed that ligand binding by the human macrophage mannose receptor, MRC1, depended on CTLD domains 4–6. We surmised that the equivalent domains of the DEC205 protein might likewise be responsible for ligand binding and might therefore be accessible to antibody in the native form of the molecule. Therefore we expressed these three domains of chicken DEC205 in a secreted Ig fusion protein (Figure S3), to provide the immunogen for raising monoclonal antibodies that might recognise chicken DEC205. From five cloned hybridomas selected after screening by ELISA, immunohistological staining and flow cytometry, two, IAH F877:FG9 (IgG1) and F877:AD6 (IgG2b) were chosen for further studies. No differences were seen between the patterns of staining with these two antibodies in innunohistochemistry or flow cytometry applications. Immunoprecipitates of unlabelled splenocyte proteins and of proteins precipitated from adherent cells after radiolabelling in the presence or absence of LPS were analysed by SDS PAGE (Figure S5). The antibody specifically precipitated a band co-migrating with the 260 kDa marker that was absent with other antibodies.

The coding sequence of chicken CD83 was identified from similar searches of EST databases as previously described [7]. The extracellular domain was expressed in the form of a soluble fusion protein with human IgG1 Fc (Figure S4) and used to raise monoclonal antibodies. Six hybridomas secreting antibody specifically recognising the CD83 part of the fusion protein in ELISA assays were cloned. These were further tested by flow cytometry for binding to chicken splenocytes, and the antibody IAH F890:GE8 (IgG2a) was selected for further use. This was shown to react with the native chicken CD83 molecule expressed on the surface of transfected COS cells (Figure S6).

### Expression of DEC205 and CD83 in tissues

**Figure 3:**
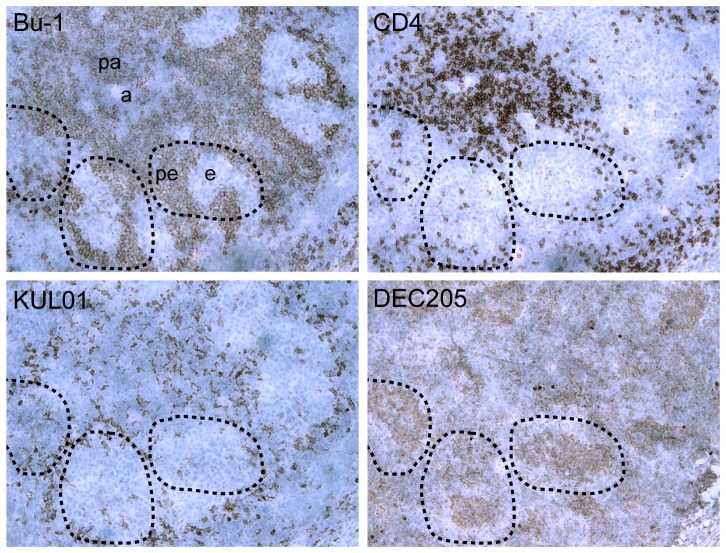
Localisation of cells expressing DEC205 in normal chicken spleen. The four panels show a set of serial sections of the same area from the normal spleen of a juvenile bird, stained with antibodies recognising the indicated molecules. Bu-1 reveals B cells and CD4 a subset of T cells. KUL01 is a marker for monocyte/macrophage lineage cells. DEC205 is detected using the antibody FG9. The dotted curves indicate the approximate outlines of the same peri-ellipsoid lymphocyte sheaths (pe) in each section. Other labelled features are ellipsoid (e), aterioles (a) and peri-arteriolar lymphocyte sheath (pa) [12].

Localization of cells expressing DEC205 in normal chicken spleen is shown in Figure 3. DEC205^+ve^ cells were principally found in the ellipsoids and immediately adjacent to arterioles, at the centre of the B cell rich (Bu1^+ve^) peri-ellipsoid lymphoid sheath (PELS) and the T cell rich (CD4^+ve^) peri-arteriolar lymphoid sheath (PALS) respectively. In contrast, macrophages (KUL01^+ve^) were found in areas delineating the borders of PELS. We did not find consistent staining with the CD83 antibody.

**Figure 4:**
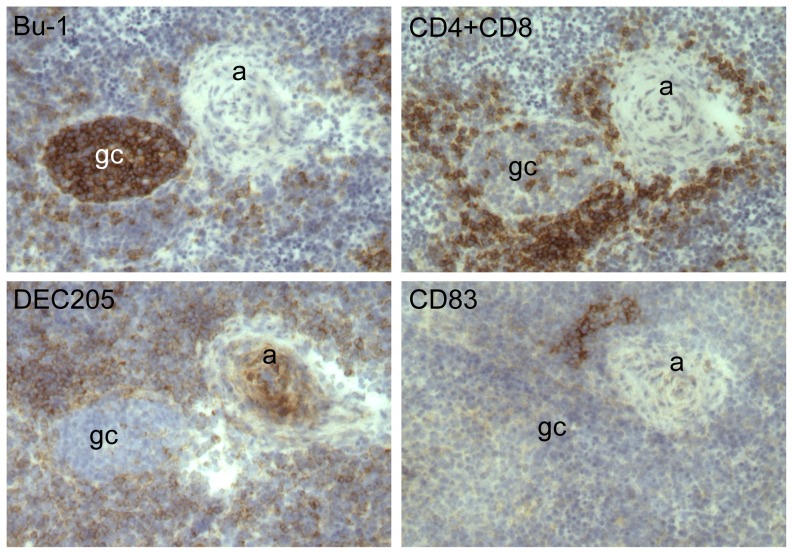
DEC205 and CD83 expressing cells in the spleen of a MDV infected bird. The four panels show a set of serial sections of the same area from the spleen of a juvenile bird five days after infection with Marek's disease virus. The sections are stained with antibodies recognising the indicated molecules. Bu-1 revealing B cells and CD4 & CD8 revealing T cells. DEC205 is detected using the antibody FG9 and CD83 using GE8. Labelled features are artery (a) and germinal centre (gc).

In the spleens of birds infected with MDV (Marek's Disease Virus), germinal centres (GC) were seen to form adjacent to arteries but within the T cell rich PALS (Figure 4). Cells expressing DEC205 were widespread within the PALS but very few were seen within the CG. CD83^+ve^ cells were found in discrete aggregates within the PALS but not within GC.

**Figure 5:**
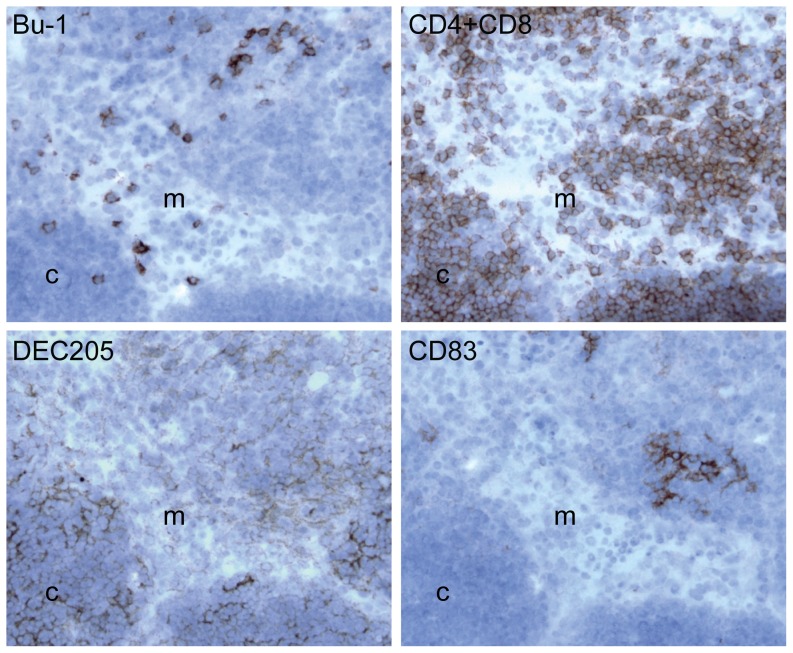
DEC205 and CD83 expressing cells in normal thymus. The four panels show a set of serial sections of the same area from the thymus of a juvenile bird. The sections are stained with antibodies recognising the indicated molecules. Bu-1 revealing B cells and CD4 & CD8 revealing T cells. DEC205 is detected using the antibody FG9 and CD83 using GE8. Cortical (c) and medullary (m) areas of a thymic lobe are labelled.

In the thymus DEC205 was seen to be expressed on cells with a reticular phenotype within both the cortex and medulla (Figure 5). Small aggregates of CD83^+ve^ cells were seen in the medulla, immediately adjacent to the cortico-medullary boundary.

**Figure 6:**
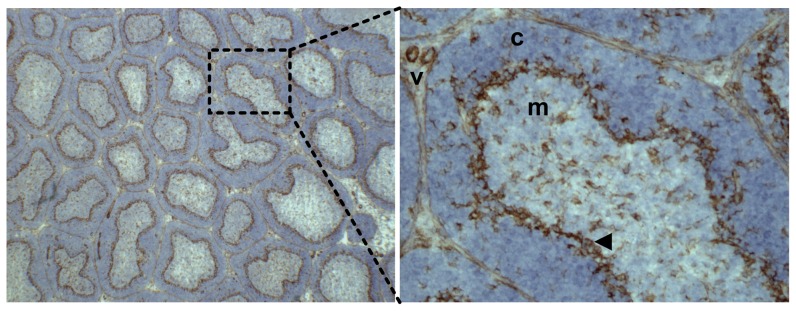
DEC205 expression in the juvenile bursa. The left panel shows a lower magnification of a section of bursal tissue stained with the DEC205 antibody FG9. The right panel shows a higher power view of the region of the same section indicated by the dashed lines. Two blood vessels in the interfolicular space are labelled (v). The cortex (c) and medulla (m) of the bursal follicle are also indicated. The arrowhead marks the layer of stained cells at the cortico-medullary boundary.

In the bursa, a continuous layer of cells at the cortico-medullary boundary strongly expressed DEC205 (Figure 6). In addition, discrete DEC205^+ve^ cells with dendritic morphology were seen evenly distributed in both cortex and medulla of the bursal follicles. We did not find consistent staining with the CD83 antibody in the bursa.

Cells in non-lymphoid tissues were also seen to express DEC205 (Figure S7). In many cases the distribution of these was consistent with expression by endothelial cells.

### Expression of DEC205 on *ex vivo* splenocytes

**Figure 7:**
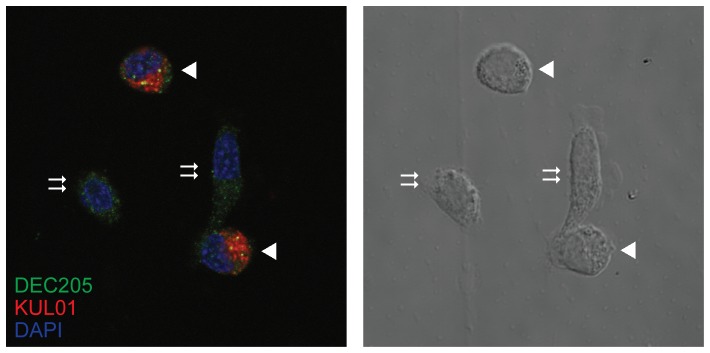
Distinct populations of adherent splenocytes expressing DEC205. Adherent spleen cells were stimulated with LPS for three hours, then permeabilised and stained with FG9 anti DEC205 antibody (green, Alexa Fluor 488) and KUL01 monocyte/macrophage marker (red, Alexa Fluor 568). Nuclei were stained with DAPI (blue). The left panel shows the pseudocolour image from the confocal microscope, and the right panel shows a differential interference contrast image of the same cells. KUL01 positive cells with rounded morphology are indicated with large arrowheads and KUL01 negative cells with flattened and extended morphology are indicated with double arrows.

**Figure 8:**
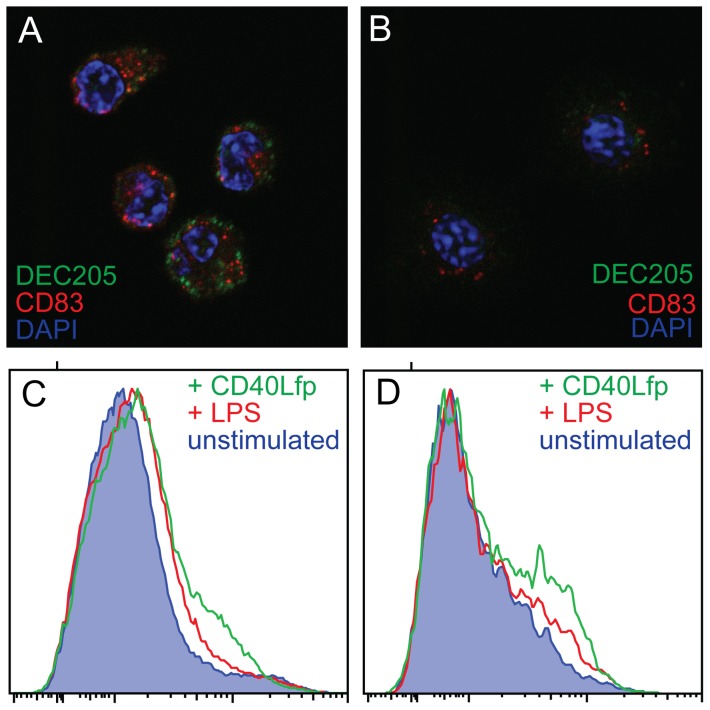
Increased expression of DEC205 on LPS stimulation of adherent spleen cells. The upper two panels show images from two chambers of the same slide, one having been incubated with LPS for one hour (A) and one without (B) The cells were stained with FG9 anti DEC205 antibody (green) and GE8 anti CD83 antibody (red). Nuclei were stained with DAPI (blue). Staining, confocal camera settings and image processing were identical for both images. Surface staining of ungated (C) or cells gated for CD11c expression (D) following stimulation with LPS or CD40 ligand fusion protein are also shown.

**Figure 9:**
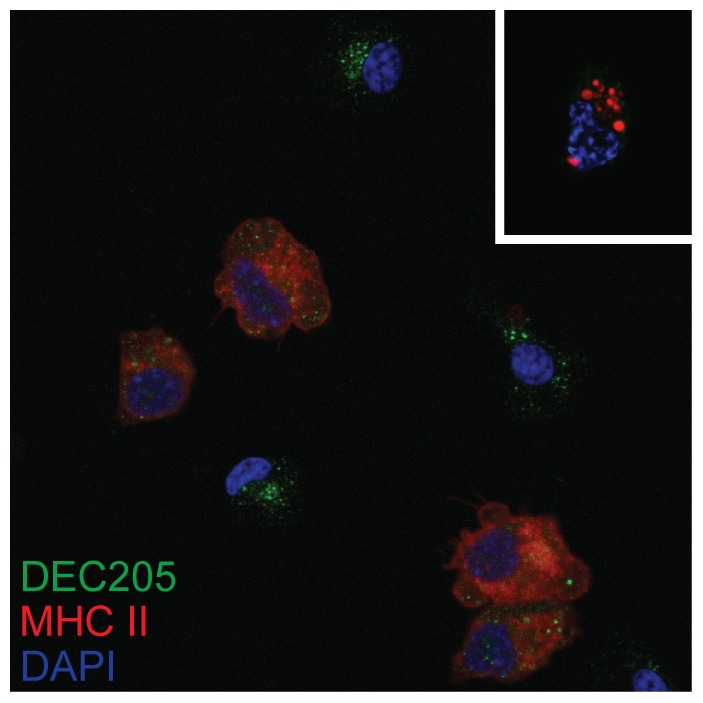
Surface mobilisation of MHC class II upon LPS activation of DEC205-expressing adherent cells. Adherent spleen cells were stimulated with LPS for three hours, permeabilised, and stained with AD6 anti-DEC205 antibody (green) and 2G11 anti MHC class II antibody (red). Nuclei were revealed with DAPI (blue). The inset shows an image of an unstimulated cell stained in the same way.

The population of cells isolated in adherent spleen cell preparations were highly depleted of lymphocytes (data not shown). Double staining of LPS-stimulated cells for DEC205 and the KUL01 antigen revealed two phenotypes (Figure 7), DEC205^+ve^, KUL01^+ve^ and DEC205^+ve^ KUL01^−ve^. Differential interference contrast imaging showed the KUL01^+ve^ cells to be macrophage-like, with condensed cytoplasm. The KUL01^−ve^ cells had more expanded cytoplasm with the appearance of veiled cells [25]. DEC205 expression was upregulated following LPS stimulation, particularly with respect to a vesicular compartment that may represent early endosomes (Figure 8A,B). CD83 expression appeared to be entirely vesicular in the unstimulated condition but rapidly increased on the cell surface following stimulation. Staining of stimulated and unstimulated cells for MHC class II (MHCII) and DEC205 is consistent with the translocation of MHC II from a lysosomal compartment to the cell surface accompanied by an up-regulation of DEC205 (Figure 9).

**Figure 10:**
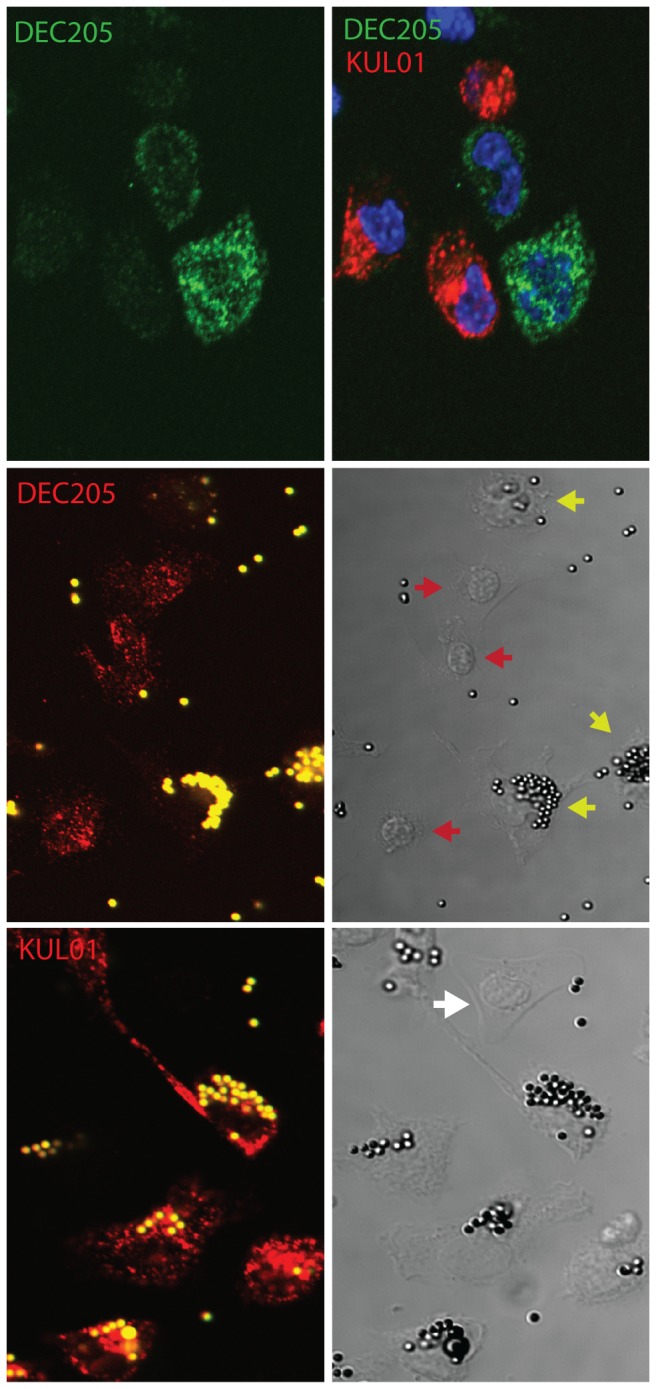
Adherent cells expressing DEC205 are non-pahgocytic. The top pair of panels shows confocal images of fresh adherent cells stained with both DEC205 and KUL01 antibodies. The left hand image reveals low level expression of DEC205 on the KUL01 positive cells. The same cell preparation was incubated with fluorescent microbeads for three hours and then fixed and permeablised before staining with either DEC205 antibody FG9 (middle) or KUL01 (bottom), and examination by confocal microscopy. The pseudocoloured fluorescence images are on the left with beads in yellow and antibody staining in red. At the right are the corresponding differential interference contrast images. Red arrows point to cells that are strongly stained with DEC205 antibody but have no internalised fluorescent beads. Yellow arrows point to cells that have internalised fluorescent beads. The white arrow points to a cell that is not stained with KUL01.

Fresh preparations of adherent cells were incubated with fluorescent microbeads to detect phagocytic activity, and then stained with either DEC205 or KUL01 antibody (Figure 10). Double staining of the fresh cells showed that DEC205 expression on KUL01^+ve^ cells was much lower than on KUL01^−ve^ cells in this preparation. Consequently the DEC205 staining of KUL01^+ve^ cells that had ingested brightly fluorescent beads was hard to discern. However, there were brightly stained DEC205 positive cells that were devoid of beads (middle) while all KUL01^+ve^ cells had ingested beads (bottom). Thus the DEC205^+ve^KUL01^−ve^ cells were not phagocytic, while the DEC205^lo^ KUL01^+ve^ cells had actively phagocytosed fluorescent microbeads.

### Expression of DEC205 on ex-vivo leucocytes

**Figure 11:**
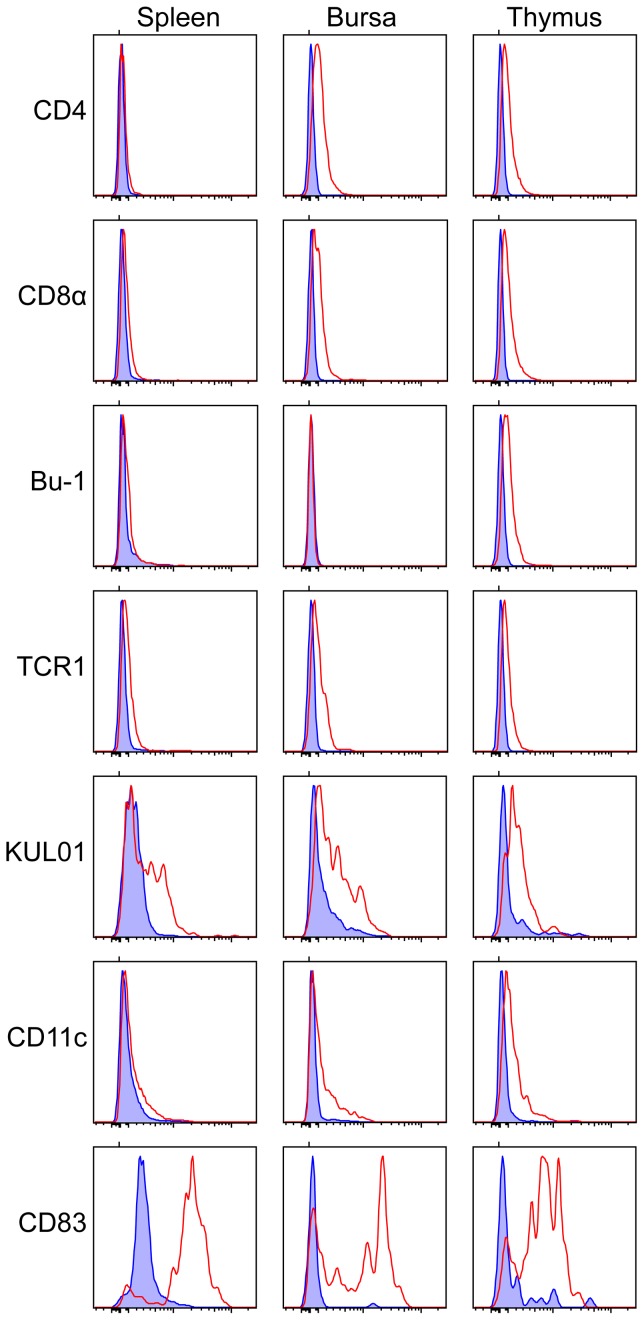
DEC205 expression on leucocyte subsets. Splenocytes, bursal cells and thymocytes recovered by enzymatic digestion were stained with DEC205 antibody and antibodies recognising each of the indicated antigens. The figure shows histograms of the DEC205 antibody fluorescence for cells gated for positive expression of the markers indicated at the left. Controls are the staining of appropriate subclass control antibodies on cells gated for the same markers.

DEC205 was expressed at very low levels on B cells and T cell subsets of leucocytes (Figure 11), although this was less evident in the spleen. Strong expression of DEC205 was seen on cells also decorated with CD83, to a level that was not apparent on KUL01+ or CD11c^+^ cells. The effect of LPS and CD40 ligand stimulation was also demonstrated by cytometry (Figure 8C,D)

## Discussion

We found that chicken DEC205 has a highly conserved gene structure and sequence with its human orthologue (EMBL AJ574899 and Figure 1). Sequence identity of 51% to human and 48% to mouse is also high for genes with immune functions from these phylogenetically distant species, suggesting selection pressure for conservation of function. The cytosolic domain of mouse DEC205 has two motifs critical to its specialist function on DC [19]. A coated pit localisation sequence FSSVRY is conserved between many species [26] and is essential for endocytosis, its removal ablating antigen uptake. The significance of a change of arginine to histidine in the corresponding chicken sequence is not clear, although this is a statistically favored substitution [27], maintaining the basic character of the replaced residue. A distal triad of acidic amino acids (EDE or DDE) is also conserved and may be critical to the targeting of antigen to late endosomes [28].

Quantitative RT-PCR indicated that the tissues most closely associated with immune responses had the highest levels of transcripts for DEC205 and CD83 (Figure 2), varying from a base level of transcription across a range of tissues. In the mouse, DEC205 transcription was only seen *in vivo* in the lymph nodes and thymus [29]. However, this was probably a consequence of the limited sensitivity of the PCR technique employed, as the monoclonal antibody NLDC-145, on which this work was based, identified individual positive cells in peripheral locations, including Langerhans cells in the skin [30]. We would surmise that the more sensitive qRT-PCR used in the present study is reporting both high levels of transcription in relatively rare cells (e.g. Langerhans cells of the skin) and the lower levels of expression we have demonstrated on other cell types (Figure 11). In contrast to our findings a previous study failed to detect any CD83 transcription in chicken skin or brain [7].

The immunization of mice by a DNA-DNA-protein prime-boost strategy facilitated the rapid production of monoclonal antibodies with specificities for DEC205 and CD83 that we then used to study the expression of these molecules *in vivo* and *ex vivo*.

The distribution of DEC205^+ve^ cells in normal spleen showed clear relationships with other immune cells and with splenic architecture (Figure 3). Particularly, DEC205^+ve^ cells were found in the ellipsoids and the immediate peri-arteriolar area, but not concentrated in the PALS or PELS. This is consistent with the distribution of ellipsoid-associated non-lymphoid cells (ENAC) [12] and ellipsoid-associated cells (EAC) [31] previously described. The location of DEC205^+ve^ and CD83^+ve^ cells during the process of germinal centre formation was studied following infection of birds with MDV (Figure 4). In this case strong expression of DEC205 on cells adjacent to arterioles was accompanied by an increase in the numbers of DEC205^+ve^ cells scattered throughout the PALS. Few DEC205^+ve^ cells were seen in germinal centres.

The ontogeny of the immune response in birds is incompletely understood, although these findings are consistent with the migration of antigen bearing EAC from ellipsoids to PALS demonstrated by other groups [31], [32]. However, the constitutive presence, in specific pathogen free (SPF) animals, of DEC205^+ve^ cells in the immediate peri-arteriolar area must now also be considered. It may be that these cells had their origin in the ellipsoids and migrated to the immediate peri-arteriolar position as the result of an undefined antigenic stimulation. The more plausible alternative is that as the central artery branches to form the pencilliar capillaries, so the immediate peri-arteriolar compartment is anatomically contiguous with the expanded peri-capilliary ellipsoids. Notwithstanding previous studies, the function of cells within the centre of the PALS with the potential for antigen uptake and presentation to T lymphocytes remains to be elucidated.

In the present study foci of cells strongly expressing CD83 were seen at the periphery of the T cell-rich PALS but not in fully formed germinal centers following infection with MDV (Figure 4). This is at variance with other work using polyclonal antibodies to CD83 [7], which concluded that CD83^+ve^ cells show a unique association with B cell compartments of the chicken.

Expression of DEC205 on thymic epithelial cells [18] is known to be involved in the clearance of apoptotic thymocytes [33], with areas of the C-type lectin domains recognising ligands associated with apoptosis and necrosis [21]. Expression in chicken thymus (Figure 5) suggests that this function is maintained. Expression of CD83 in chicken thymus is restricted to foci of cells within the medulla but adjacent to the cortico-medullary junction. Although these appear similar to the DC foci induced around Hassall's Corpuscles, that are responsible for the induction of CD4^+ve^CD25^+ve^ regulatory T cells in human thymic medulla [34], further work is required to confirm this relationship.

Expression of DEC205 in the bursa is more complex (Figure 6). Its distribution closely resembles that of vimentin expressing BSDC previously described by Olah and Glick [35]–[37]. A minor population of DEC205^+ve^ cells with dendritic morphology are dispersed throughout both medulla and cortex. In contrast to this scattered distribution, at the cortico-medullary boundary there is a continuous layer of DEC205^+ve^ cells. A role for BSDC in driving gene conversion and the selective expansion of the B cell compartment, has been proposed [38]. The relevance of the separation of cortex and medulla by cells expressing DEC205 is unclear. It has been proposed that B cell migration from the cortex to medulla might be induced by encountering gut antigens. While the dispersed BSDC are ideally situated to initiate migration between these compartments, the continuous layer at the boundary could enable more stringent regulation of the process. DEC 205 has been shown to be a receptor for apoptotic self [21] and to be involved in the clearance of apoptotic thymocytes [33]. DEC205^+ve^ cells in the bursa may have a similar role, as the majority of bursal cells generated each day do not emigrate but die *in situ* [39] by the mechanism of apoptosis [40].

The analysis of *ex-vivo* avian DC has not hitherto been reported, although DC induced by cytokines *in-vitro* have been studied [3]–[5]. We used short term adherence to enrich for a population of splenocytes that were depleted of lymphocytes but had the morphological characteristics of DC and macrophage. Without prior stimulation only cells that expressed the KUL01 antigen were able to phagocytose microbeads, while cells expressing high levels of DEC205 were not phagocytic. Although both cell types could upregulate DEC205 expression following LPS stimulation, the KUL01 antigen remained restricted to macrophage–like cells while DEC205^+ve^ cells retained classical DC morphology. These cells were also able to mobilise MHC class II to the cell surface. We propose a working definition of the unstimulated *ex-vivo* avian non-phagocytic DC as being DEC205^+ve^, KUL01^−ve^, and expressing MHC class II in a vesicular compartment.

Low level expression of DEC205 on leucocytes other than DC was first described on mouse lymph node B cells [41] and has subsequently been shown on human B and T lymphocytes, NK cells and macrophages [42]. Here we report low-level expression on chicken CD4^+ve^, CD8^+ve^ and γδ T lymphocytes, B lymphocytes and macrophages, the relative expression being dependent on the tissue origin of the cells. The function of DEC205 on these cells remains a matter of speculation.

In summary we have sequenced chicken DEC205, and produced monoclonal antibodies with specificity for DEC205 and CD83. We have identified the populations of cells expressing DEC205 in the major immune organs and determined expression on ex-vivo cells, allowing us to propose a phenotypic definition of avian DCs, as DEC205^+ve^ and KUL01^−ve^ that now needs to be expanded and tested in functional studies. The many similarities with mammalian species are balanced by the differing requirements of the unique architecture of the avian immune system. In particular, the identification of a layer of DEC205 expressing cells at the cortico-medullary junction of the bursa points to a novel function of this molecule in the physiology of this primary lymphoid organ.

## Materials and Methods

### Ethics Statement

All animal procedures were performed in accordance with the UK Animals (Scientific Procedures) Act 1986 [43]. This study was approved by the Pirbright Institute Ethical Review Panel and the UK Home Office under project licence 30/2683.

### Experimental animals

RPRL (Regional Poultry Research Laboratory, East Lansing, MI.) Line 6 and Line 7 birds were obtained from the Compton specific pathogen free breeding facility, from parents negative for antibodies to specified pathogens, and were kept in controlled-environment isolation rooms with food and water provided *ad libitum*.

For DC and macrophage preparations, spleens were collected, from Line 6 or Line 7 birds between 2 and 10 weeks old, into sterile PBS on ice. For RNA preparations, tissue sections (approximately 50 mg) were collected into RNA Later stabilization fluid (Ambion, UK).

Two week old line 7 birds were infected with Marek's disease virus (MDV) strain RB1B, by administering 2.5 mg infective dust by the intra-tracheal route [44]. Tissue samples from infected and control birds, killed by cervical dislocation at various times after infection, were used for cryosectioning and immunostaining.

### Cell lines

Cell cultures were maintained in 5% CO_2_ at 37°C. COS-7 cells [45] were grown in Dulbecco's Modified Eagles Medium (DMEM) (Invitrogen) with Glutamax, supplemented with 100 U/ml Penicillin, 100 µg/ml streptomycin and 10% FCS. CHO cells [46] [47] were grown in Ham's F12 medium (Invitrogen) with 10% FCS. Puromycin HCl (Enzo) was used at 20 µg/ml for selection and at 15 µg/ml for maintenance of transfected CHO cells.

### Antibodies

All antibodies used and their sources are described in Table S1.

### PCR and sequencing of DEC205 gene

Bursa and spleen cDNA libraries were used for PCR-based cloning of chicken cDNA. The bursa library was derived from cDNA of 17-day embryonic bursa from Light Sussex chickens, cloned in pCDM8 [48]. The spleen library was made from Line 0 chicken spleen cDNA, cloned in pcInX [49]. The vector pcInX is pCINeo (Promega) with the *XbaI-NotI* fragment in the multiple cloning site replaced by the *XbaI-NotI* fragment from pCDM8 (Invitrogen). It has two mutually inverted *BstXI* cloning sites. A single *BstXI* site in the 3′UTR of the Neo gene was removed by cutting pcIneo with *BstXI*, treating with Klenow fragment of DNA polymerase and re-ligating, before the modifications to the cloning site [50]). Cloning of cDNA into this vector, or into pCDM8, was carried out using the *BstXI* linker method described by Aruffo and Seed [51].

PCR reactions were carried out using Taq polymerase (Invitrogen) using the manufacturer's recommended conditions with 1.5 mM MgCl_2_, 0.2 mM dNTP (Promega), 1 µM primers and 2.5 U of Taq polymerase (Invitrogen Life Technologies) in a final volume of 50 µl with 50 ng of library plasmid DNA. PCR was performed in a Biorad thermocycler as follows: 94°C for 2 min followed by 30 cycles of 94°C for 30 sec, 55°C for 30 sec, 72°C for 60–120 sec and a final 10 min extension at 72°C. Amplified products were recovered from agarose gels (Qiagen QIAquick gel extraction kit) and cloned into pGEM-T (Promega). DNA prepared by Qiagen QIAprep spin miniprep kit was used for sequencing.

A chicken BAC library (UK HGMP Resource Centre) was screened by hybridisation with amplified and cloned DEC205 cDNA to identify 9 DEC205 positive BACS. One of these (103-N18) was selected for sequence analysis, performed by primer walking, from exon 2 to the 5′ end of the coding sequence. Sequencing was carried out directly on BAC DNA template purified by Nucleobond BAC 100 maxiprep kit (Macherey-Nagel).

All DNA sequencing reactions were performed using the Quickstart kit (Beckman Coulter) and were analysed using the Beckman Coulter CEQ8000 DNA sequencer. Sequence data were analysed using STADEN [52] and GCG (Wisconsin Package Version 10.2, Genetics Computer Group, Madison, WI). Multiple clones of PCR products were sequenced from each amplification to obtain the consensus sequence and to identify clones free from PCR errors.

### Generation of monoclonal antibodies

An immunogen for production of DEC205 monoclonal antibodies was produced by PCR amplification of cDNA encoding C-type lectin-like domains (CTLD) 4 to 6 and insertion of the amplified sequence in frame between a mouse CD8α signal peptide and a human IgG1 constant region in which the hinge cysteine residues had been replaced by serine residues [53], in the vector pcIpac. This was accomplished by replacing the *Nhe* I – *Bgl* II fragment, encoding the extracellular domain of chicken CD86, of a construct, pKW06, expressing chicken CD86-human-IgG1Fc (JRY and Kathryn Wright, unpublished). Primers used and the confirmed junction sequences are described in the data (Figure S3). After transfection of this construct into CHO cells using Effectene (Qiagen) and selection with puromycin, transfected cells were cloned by limiting dilution. Clones were tested for expression using a sandwich ELISA for human IgG Fc using unlabelled and HRP labelled goat anti-human IgG (Southern Biotech) as capture and detection reagents. The fusion protein was purified from high density culture (miniPERM, Sarstedt) supernatants by binding to protein G columns (GE Biosciences, Hi-Trap), washing with PBS, and elution with 0.1 M Glycine HCl, pH 2.7, according to the manufacturer's instructions for antibody purification. After dialysis against PBS, the concentration of protein was estimated from absorbance at 280 nm using a conversion factor (molar extinction coefficient, 162480) calculated from the peptide sequence (http://web.expasy.org/protparam/).

While these manipulations were being conducted, mice were immunized with two intramuscular injections of the fusion protein expression plasmid at four week intervals. 100 µg of plasmid (endotoxin free, Qiagen Endofree Plasmid Maxi Kit) was injected intramuscularly in PBS. After a further four weeks, mice received a final boost with intraperitoneal injection of 50 µg purified fusion protein and were sacrificed four days later for preparation of splenocytes which were fused with NS0 hybridoma partner cells using established methods. Hybridoma supernatants were first screened by ELISA for antibodies binding to fusion protein immobilised with anti-human IgG, detected with HRP conjugated goat anti-mouse IgG. Antibodies recognising the human Ig moiety of the fusion protein were eliminated by a similar ELISA using a control fusion protein containing the same human IgG1 sequence. This was followed by flow cytometry using leucocytes prepared from spleens by enzymatic digestion, and by immunostaining of spleen, bursa and thymus sections using the VECTASTAIN Elite ABC kit (Mouse IgG) (Vector laboratories) to detect binding of the primary antibody.

For CD83 antibodies, a similar strategy was employed, using a fusion protein in which the entire extracellular domain of chicken CD83 was fused to the same human IgG1 Fc region. Details of the primers used for amplification and introduction of restriction sites for insertion into pKW06 are described in the supplementary data (Figure S4). The cDNA was amplified from a ConA-stimulated splenocyte library described previously [54]. COS-7 cells were transfected using COSFectin (BioRad) following manufacturer's instructions. The fusion protein was purified from the supernatant of COS-7 cells transfected with the constructed plasmid. For detection and cloning of hybridomas, supernatants were screened by sandwich ELISA for reactivity with the CD83 fusion protein but not with a control Ig fusion protein.

### Quantitation of DEC205 and CD83 transcripts

RNA was extracted from 30 mg samples of tissues using the Nucleospin RNA II kit (Macherey-Nagel) according to the manufacturer's instructions. Homogenisation was performed using a Mixer Mill MM300 (Retsch) and 3 mm stainless steel cone balls (Retsch). The RNA isolation kit includes a DNase digestion step. An additional proteinase K digestion was performed on heart, muscle, skin and lung tissue to aid removal of contractile tissue, connective tissue and collagen by addition of >6 mAU Proteinase K (Qiagen) to the homogenised lysate and incubating at 55°C for 10 min.

Primers and probes for real-time quantitative PCR assay (Taqman®; Applied Biosystems, Foster City, California, USA) were designed using Primer Express software (Applied Biosystems) (Table S3). Probes were labelled with the fluorescent reporter dye 5-carboxyfluorescein (FAM) or 5-Yakima Yellow at the 5′ end and the quencher N,N,N,N′ tetramethyl-6-carboxyrhodamine (TAMRA) at the 3′ end. Assays were carried out using the Superscript III platinum one-step qRT-PCR kit (Invitrogen). Amplification and detection of specific products were carried out with the 7500 Fast Real Time System (Applied Biosystems) with the following cycle profile: 50°C for 5 min, 95°C for 2 min and then 40 cycles of 95°C for 3 sec and 60°C for 30 sec. The reference used for standardization was 28 s RNA. Primer and probe sequences are presented in Table S3. Data analysis was performed as described previously [55]. This yields transcript levels in terms of a number log_2_R_0_, on a logarithmic (base 2) scale where the value 0 represents the lowest detectable level of the target mRNA in the sample with the most reference (28S) RNA, and positive numbers represent the log_2_ of the fold increase over that value in the test samples. The calculation includes a correction for different levels of reference RNA in each sample.

### Preparation of cell suspensions

Leucocytes were prepared from spleens by enzymatic digestion. Spleens were washed in PBS and injected with a total of 3–4 ml of 2.2 mg/ml collagenase D (Boehringer Mannheim) in HBSS (Sigma). After incubation at 37°C for 15 min in a shaking incubator, cell suspensions were transferred to cold 10 mM EDTA in HBSS without calcium (Sigma). Digestions were repeated until the spleen tissue had completely disintegrated. The collected cell suspension was filtered using a 100 µm cell strainer (BD Falcon), centrifuged at 220×g for 10 minutes at 4°C, resuspended in RPMI 1640 medium with Glutamax (Invitrogen) supplemented with 5% FCS (Invitrogen). The suspensions were layered onto HistoPaque 1119 (Sigma), centrifuged at 2000 rpm (492×g) for 20 min at 4°C, washed, and resuspended at 5×10^6^/ml in RPMI 1640 containing 10% FCS.

### Isolation of adherent cells

Either 5×10^6^ cells per well in 24-well plates containing a 13 mm glass coverslip, or 2.5×10^6^ cells per chamber of an 8-chamber slide (Lab-Tek II), were incubated at 37°C for 2 h. The non-adherent cell population was discarded and the adhered cells subjected to a vigorous wash, then further cultured in RPMI 1640 containing 10% FCS. The adherent cell population was incubated either alone or with addition of LPS (Sigma) at 200 ng/ml for various times before fixation with 4% paraformaldehyde for 20 min. To observe phagocytosis, the enriched population was incubated with 1 µM yellow-green carboxylate-modified microspheres (Invitrogen) for 3 h, and observed using a confocal microscope (Leica SP2 with 405-, 488-, and 568-nm lasers).

### Immunostaining of tissue sections and adherent cell populations

Tissues were collected into Cryosection Tissue Reservoirs surrounded by OCT medium (Tissue Tek) and snap frozen in a freezing bath of iso-pentane (2-methylbutane) on dry ice. Tissues sections (6 mm) were cut using a cryostat (Leica CM1900) and collected onto Polysine coated slides, air-dried overnight, and fixed in ice cold 80% acetone, 20% methanol for 10 min. Slides were stored in airtight containers at −70°C. Thawed sections were incubated for 60 min with antibodies that were diluted to 5 µg/ml in 0.4% (w/v) BSA in PBS. After washing with PBS, bound antibodies were visualised using the Vectastain Elite ABC kit (Vector Laboratories), and the Vector NovaRed substrate kit (Vector Laboratories), according to the manufacturer's instructions. Sections were counterstained with haematoxylin (Gill no. 3; Sigma) and permanently mounted in Clearium (Surgipath).

For confocal imaging, adherent cells prepared as above were permeabilised by incubation with 0.1% triton X-100 for 15 min, after which non-specific reactivity was blocked by incubation with 5% (v/v) normal goat serum in PBS for 60 min. This was followed by 60 min incubation with 5 µg/ml primary antibody in blocking solution. Bound antibodies were detected using Alexa Fluor labelled IgG class-specific antibodies (Table S1). Nuclei were stained by incubation with 2 µg/ml DAPI (Sigma) for 10 min. Sections were mounted in Vectashield (Vector Laboratories) and analyzed using a confocal microscope as above.

### Flow Cytometry

Cells were prepared as above. Immunofluorescent labeling was carried out using DEC205 primary antibody (*FG9*) conjugated to Alexa *Fluor* 647 and CD83 antibody conjugated to Alexa *Fluor* 488 in combination with antibodies to KUL01, Bu-1, TCR-1, CD4, CD11c or CD8β conjugated to PE. Doublets were excluded by analysis of side scatter height and area parameters and dead cells by LIVE/DEAD® Fixable *Aqua* (Invitrogen).

For all labeling steps, cells (1×10^6^) were incubated for 15 min at room temperature with appropriate dilutions of antibody in U bottom 96-well microtitre plates with two washes between each step. Normal mouse serum (5%) was used to block non-specific binding of antibodies. PBS containing 1% BSA and 0.1% sodium azide was used as dilution and washing buffer. Cells were analysed using a FacsCalibur instrument (BD Becton Dickinson). FlowJo software (TreeStar) was used to analyse flow cytometry data.

## Supporting Information

Figure S1 **PCR primers and products used to determine sequences of chicken DEC205 cDNA.**
(PDF)

Figure S2 **Sequencing primers used to determine sequences of the 5′ end of the chicken DEC205 gene from BAC DNA.** The primer sequences are provided in table S2.
(PDF)

Figure S3 **Structure of chicken DEC205-human IgG-Fc fusion.**
(PDF)

Figure S4 **Structure of the CD83-human IgG1-Fc fusion protein.**
(PDF)

Figure S5 **DEC205 antibody FG9 precipitates 260 kDa native protein.**
(PDF)

Figure S6 **GE8 monoclonal antibody recognises the chicken CD83 gene product expressed on transfected COS cells.**
(PDF)

Figure S7 **DEC205 expression in non-lymphoid tissues.**
(PDF)

Table S1 **Antibodies.**
(PDF)

Table S2 **Primers used for determining the chicken DEC205 sequence.**
(PDF)

Table S3 **Quantitative reverse transcription-PCR primer and probe sequences.**
(PDF)
