# Supplementary material for: Expression of Chicken DEC205 Reflects the Unique Structure and Function of the Avian Immune System
Source: PLoS One. 2013 Jan 9;8(1):e51799. doi: 10.1371/journal.pone.0051799 (PMC3541370; doi:10.1371/journal.pone.0051799)
Supplement: Figure S1 — PCR primers and products used to determine sequences of chicken DEC205 cDNA. (PDF) [file pone.0051799.s001.pdf]

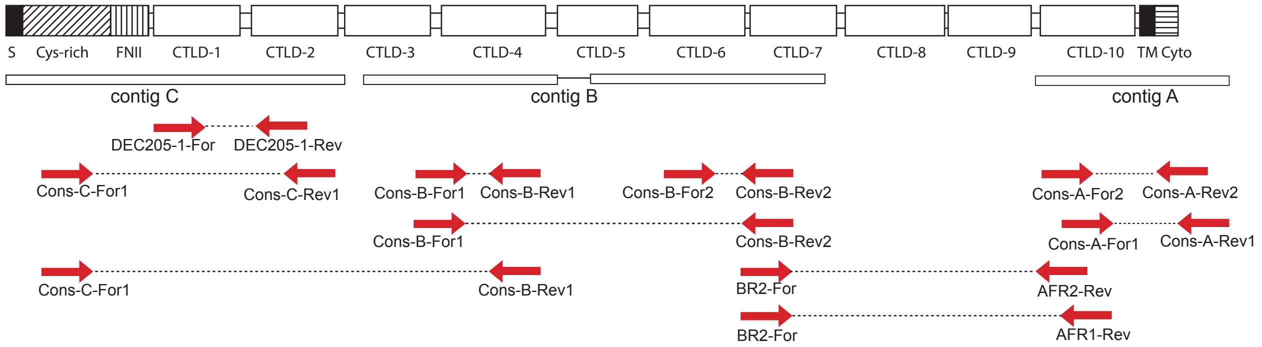

Supplementary figure S1. PCR primers and products used to determine sequences of chicken DEC205 cDNA. Bars labelled contig A-C show the regions covered by available EST data. These were used to design the indicated primers for amplification of cDNA. The dotted lines show the PCR products from which multiple clones were sequenced. The sequences of the primers are provided in table S2.
