# Supplementary material for: Expression of Chicken DEC205 Reflects the Unique Structure and Function of the Avian Immune System
Source: PLoS One. 2013 Jan 9;8(1):e51799. doi: 10.1371/journal.pone.0051799 (PMC3541370; doi:10.1371/journal.pone.0051799)
Supplement: Figure S2 — Sequencing primers used to determine sequences of the 5′ end of the chicken DEC205 gene from BAC DNA. The primer sequences are provided in table S2. (PDF) [file pone.0051799.s002.pdf]

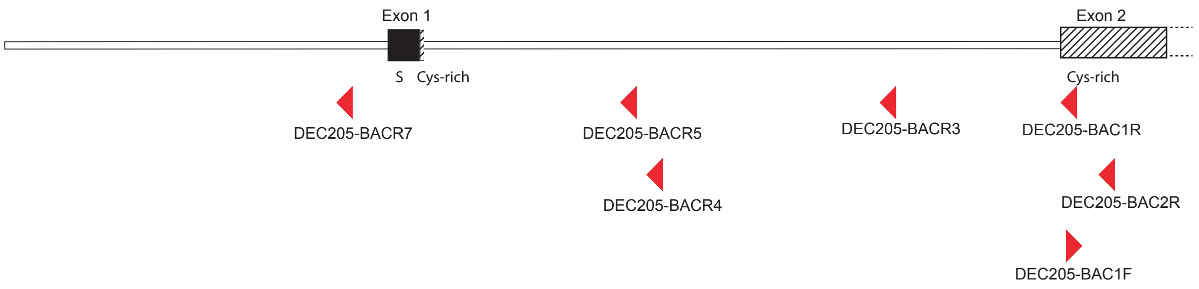

Supplementary figure S2. Sequencing primers used to determine sequences of the 5' end of the chicken DEC205 gene from BAC DNA. The primer sequences are provided in table S2.
