# Supplementary material for: Expression of Chicken DEC205 Reflects the Unique Structure and Function of the Avian Immune System
Source: PLoS One. 2013 Jan 9;8(1):e51799. doi: 10.1371/journal.pone.0051799 (PMC3541370; doi:10.1371/journal.pone.0051799)
Supplement: Figure S3 — Structure of chicken DEC205-human IgG-Fc fusion. (PDF) [file pone.0051799.s003.pdf]

```
                                                                              NheI
                                                                     CTCCCAAAGGCTAGCGATCCCTGTCC-> (DEC205F-NheI)
ATGGCCTCACCGTTGACCCGCTTTCTGTCGCTGAACCTGCTGCTGCTGGGTGAGTCGATTATCCTGGGGAGTGGAGAAGCTAGCGATCCCTGTCCCCCTGGGTGGCATAATGGGTCTGGC
 M  A  S  P  L  T  R  F  L  S  L  N  L  L  L  L  G  E  S  I  I  L  G  S  G  E  A  S  D  P  C  P  P  G  W  H  N  G  S  G
<-Mouse CD8α signal peptide--------------------------------------------------------->    <-CTLD4----------------------

CTTGCCTGCTACAAGTTCTTCCACAGTGAAAGAGTGCTGCGCACCAGAACTTGGGAGGAGGCGGAAAGGTTCTGTGAAGCCCTCGGAGGCCATCTTCCCAGCTTCACTCATACAGAAGAA
 L  A  C  Y  K  F  F  H  S  E  R  V  L  R  T  R  T  W  E  E  A  E  R  F  C  E  A  L  G  G  H  L  P  S  F  T  H  T  E  E
-CTLD4------------------------------------------------------------------------------------------------------------------

ATCAAGACGCTTCATTCCATCCTGAGAAAAATTATCAGTAATGACAGATGGGTATGGGTTGGAATGAATAAGAGGAGTCCAGATTCTCTGGGAACCTGGCAATGGAGTGACGATAAACCC
 I  K  T  L  H  S  I  L  R  K  I  I  S  N  D  R  W  V  W  V  G  M  N  K  R  S  P  D  S  L  G  T  W  Q  W  S  D  D  K  P
-CTLD4------------------------------------------------------------------------------------------------------------------

GTAACCAGTCTTGTCTTGCCACATGATTATCTGGAAGACGAGTATGACACAAGGGACTGTGTTGCTTTAAAGACCTTTCAGTTTTCACGGAGATCATTTTGGAGGTTTTATTTCCATGAA
 V  T  S  L  V  L  P  H  D  Y  L  E  D  E  Y  D  T  R  D  C  V  A  L  K  T  F  Q  F  S  R  R  S  F  W  R  F  Y  F  H  E
-CTLD4------------------------------------------------------------------------------------------------------------------

GGCAGAGACCTGGAGTTTTATTTCAAGCCTTTTGATTGTGAGGCTAAACTTGAATGGGTCTGCCAGATAACTAAAGGTAGCACTCCAAAGACACCTGAGTGGTATATACCAGATGAAATT
 G  R  D  L  E  F  Y  F  K  P  F  D  C  E  A  K  L  E  W  V  C  Q  I  T  K  G  S  T  P  K  T  P  E  W  Y  I  P  D  E  I
-CTLD4------------------------------------------------------------->

GGAATTCATGGAGTCCCACTTGTTGTTGATGGAGCGGAGCTGTGGTTTGTACCAGATAAAAACGTGAGCTTTCAAGAAGCTATTTCCTACTGTCAGAAAAACGATAGTGAATTGGCCTCT
 G  I  H  G  V  P  L  V  V  D  G  A  E  L  W  F  V  P  D  K  N  V  S  F  Q  E  A  I  S  Y  C  Q  K  N  D  S  E  L  A  S
   <-CTLD5--------------------------------------------------------------------------------------------------------------

GTGGAGTCTTACCCAAAACTCAGGACAATACTTTCTCAGATAGAAAAGTTATCAAACAGTGAACAGAAGTGGTGGCTGAAGTTTATTGATTACGGCTACAGCTATCATTCACCTTTACAG
 V  E  S  Y  P  K  L  R  T  I  L  S  Q  I  E  K  L  S  N  S  E  Q  K  W  W  L  K  F  I  D  Y  G  Y  S  Y  H  S  P  L  Q
-CTLD5------------------------------------------------------------------------------------------------------------------

TTATTTCCACGCTTCCATGATCGATCCCTGAGGGACTGCTGGTATGTTTCTAGGAAGAGCTGGTATAGAGACTACCCAGTGAACTGTAACATGAAGCTGCCCTTCATTTGTGAGAAGAAT
 L  F  P  R  F  H  D  R  S  L  R  D  C  W  Y  V  S  R  K  S  W  Y  R  D  Y  P  V  N  C  N  M  K  L  P  F  I  C  E  K  N
-CTLD5---------------------------------------------------------------------------------------------------------->

AATGCCTCCTTGCTGGAGAAACACGATCCCAGTTACCGCCCGGTCACAGGAGGTTGCCCCAAGGGTTGGCTTCGATTTCGGAATAAGTGCTTCCTAAAGATGAAATCTGAGTATTTAACA
 N  A  S  L  L  E  K  H  D  P  S  Y  R  P  V  T  G  G  C  P  K  G  W  L  R  F  R  N  K  C  F  L  K  M  K  S  E  Y  L  T
                                                           <-CTLD6------------------------------------------------------

TTCAATGCAGCGAATGAGAAGTGTGTAACTTTTGGAGGCTCTCTTCCATGCATCTCAAGTCAAGCTGAGCAAGATTTTATAACATCCTTGCTTCCTCAAATGCCAAGAGATATTTGGATT
 F  N  A  A  N  E  K  C  V  T  F  G  G  S  L  P  C  I  S  S  Q  A  E  Q  D  F  I  T  S  L  L  P  Q  M  P  R  D  I  W  I
-CTLD6------------------------------------------------------------------------------------------------------------------

GGTTTGCAGTTTTTGTTCAGCACGAGAGAAAACAAGTGGATAGATGAGAGCAGACTGTTATATAGTAACTTTCACCCACTCCTGACAGGAAGACTGAGAAAGATTCCACTGGATCTGTTT
 G  L  Q  F  L  F  S  T  R  E  N  K  W  I  D  E  S  R  L  L  Y  S  N  F  H  P  L  L  T  G  R  L  R  K  I  P  L  D  L  F
-CTLD6------------------------------------------------------------------------------------------------------------------

GATGAAGAATTTAACAATCAGTGTGGTGTAATCCTCAATGATCCCAAGTCTCACTATGTTGGAACATGGAATTTCACTGCTTGCGCTGACAGGCACTTCTTGGGTATATGCCAGCGTCCT
 D  E  E  F  N  N  Q  C  G  V  I  L  N  D  P  K  S  H  Y  V  G  T  W  N  F  T  A  C  A  D  R  H  F  L  G  I  C  Q  R  P
-CTLD6---------------------------------------------------------------------------------------------------------->

                 Bgl II
     <-TCCTCGACGTCTAGAGGTCTGT (DEC205R-BglII)
ATAGGTATAGGAGCTGCAGATCTGGAGCCCAAATCTTCTGACAAAACTCACACATCCCCACCGTCCCCAGCACCTGAGCTCCTGGGGGGATCGTCAGTCTTCCTCTTCCCCCCAAAACCC
 I  G  I  G  A  A  D  L  E  P  K  S  S  D  K  T  H  T  S  P  P  S  P  A  P  E  L  L  G  G  S  S  V  F  L  F  P  P  K  P
                 <-human IgG1 Fc----------------------------------------------------------------------------------------
```

Supplementary figure S3. Structure of chicken DEC205-human IgG-Fc fusion. The coding sequence including that for the mouse CD8α signal peptide is shown above the encoded peptide sequence. The coloured dashes below indicate protein domains, the signal sequence (red), the three chicken DEC205 CTLD domains, and the start of the human IgG1 Fc (black). The codons and encoded serine residues in blue and boxed are those replacing cysteine residues in the hinge region of the original Fc sequence. The sequences of the primers used to amplify that part of the chicken DEC205 cDNA used in the construct are shown above the cDNA sequence, with the restriction sites used for construction boxed.
