# Supplementary material for: Expression of Chicken DEC205 Reflects the Unique Structure and Function of the Avian Immune System
Source: PLoS One. 2013 Jan 9;8(1):e51799. doi: 10.1371/journal.pone.0051799 (PMC3541370; doi:10.1371/journal.pone.0051799)
Supplement: Figure S4 — Structure of the CD83-human IgG1-Fc fusion protein. (PDF) [file pone.0051799.s004.pdf]

```
                  Nhe I
          TGATTTGAGCTAGCAATACCCATG-> (CD83F-NheI)
GCTGCTCTGTGATTTGAAGCAGCAATACCCATGGCTTCAGCAGCCTACACTCTACTCTTCACCCTGTGCAATGTTTGGAGCTTGATCAATGGAGCTGCTGTGGCTGTCCCAGATGTTGCT
                               M  A  S  A  A  Y  T  L  L  F  T  L  C  N  V  W  S  L  I  N  G  A  A  V  A  V  P  D  V  A

GTGACATGCTTTGAAGAAGCTTTGCTGTCCTGTAAAGTTCTTCAGGATTCCTCGATCGCCTACCAGGCAGTGTCTTGGCACAAAATGGCTGGAGTTGGCGACAGAATAGCATGGAAAGTC
 V  T  C  F  E  E  A  L  L  S  C  K  V  L  Q  D  S  S  I  A  Y  Q  A  V  S  W  H  K  M  A  G  V  G  D  R  I  A  W  K  V

CTTGATGTGGAATCTCGTCATCCAAAAGGACTTGGAGGCTCCCTGGAGCTCTCCAACACCACCTTTCAACTGAGGATCAGGAATGCCACCAGCCAGGACAGTGGCACGTACAAGTGCGCT
 L  D  V  E  S  R  H  P  K  G  L  G  G  S  L  E  L  S  N  T  T  F  Q  L  R  I  R  N  A  T  S  Q  D  S  G  T  Y  K  C  A

                                                                                                     Bgl II
                                                                         (CD83R-BglII)   <-TATGTTCTGTCTAGAAAAGTACGA
TTGGGGAACAGAGGGGAGACCACAACCTGAGTGGCATCATCACATTAAAAGTAACAGGTTGCCCTAGAATAGAAGATGAAAAACTGAAAAAAATACAAGACAGATCTGGAGCCCAAATCT
 L  G  E  Q  R  G  D  H  N  L  S  G  I  I  T  L  K  V  T  G  C  P  R  I  E  D  E  K  L  K  K  Y  K  T  D  L  E  P  K  S
```

Supplementary figure S4. Structure of the CD83-human IgG1-Fc fusion protein. The coding sequence is shown above the encoded peptide sequence. The signal peptide predicted by SignalP 3.0 is coloured red. The primers used in the construction are indicated above the sequence and the restriction sites they introduced for construction are boxed.
