# Supplementary material for: Expression of Chicken DEC205 Reflects the Unique Structure and Function of the Avian Immune System
Source: PLoS One. 2013 Jan 9;8(1):e51799. doi: 10.1371/journal.pone.0051799 (PMC3541370; doi:10.1371/journal.pone.0051799)
Supplement: Figure S5 — DEC205 antibody FG9 precipitates 260 kDa native protein. (PDF) [file pone.0051799.s005.pdf]

Supplementary figure S5.

Immunoprecipitates of unlabelled splenocyte proteins and of proteins precipitated from adherent cells after radiolabelling in the presence or absence of LPS were analysed by SDS PAGE (Figure S5). The antibody specifically precipitated a band, co-migrating with the 260 kDa marker, that was absent with other antibodies. A labelled band with the same apparent molecular weight was specifically precipitated by FG9 from LPS treated adherent cells and was not precipitated by a control mAb or other antichicken antibodies. Without LPS treatment, this band was either much less abundant or absent.

The human DEC205 orthologue, which has a peptide molecular weight of 195 kDa and 14 predicted (1) sites for N-glycosylation, migrates on SDS PAGE with an apparent molecular weight of 250 kDa (2, 3). The peptide molecular weight of the protein encoded by the chicken DEC205 gene is 197 kDa, and there are 15 predicted N-glycosylation sites. Therefore the observed 260 kDa apparent molecular weight of the precipitated molecule is consistent with its being the product of this gene. The increase in quantity of the protein in LPS treated adherent cells is consistent with the increase in fluorescent staining in confocal microscopy using this antibody.

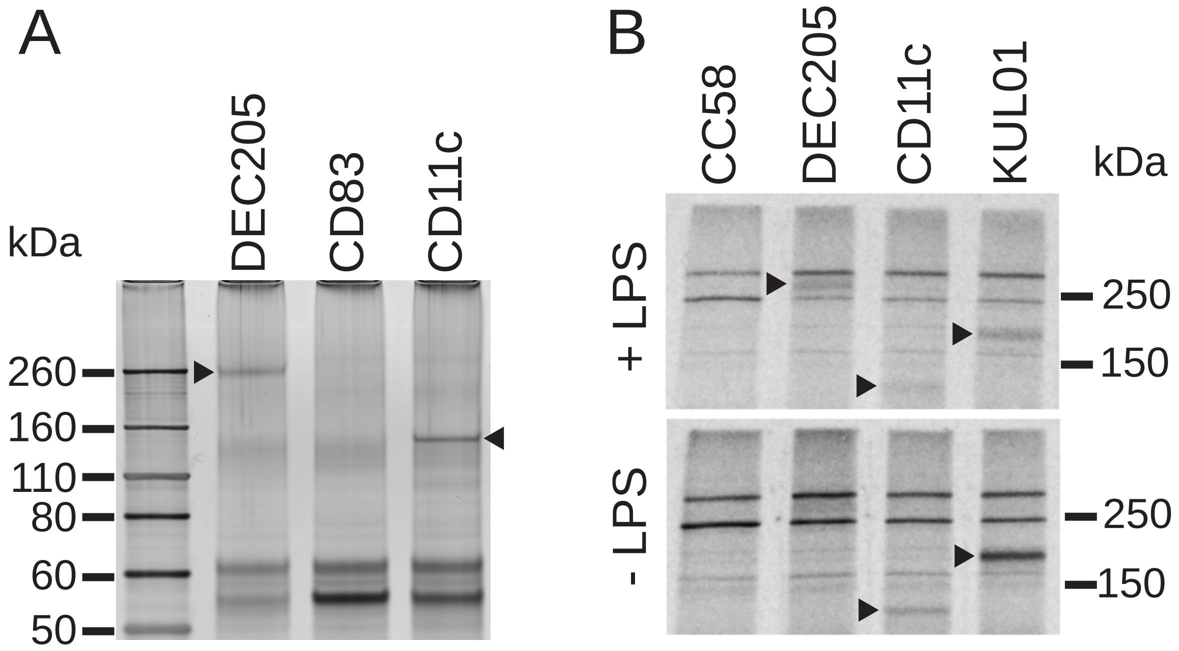

Supplementary figure S5. DEC205 antibody FG9 precipitates 260 kDa native protein.

(A) Silver stained SDS-PAGE gel of proteins immunoprecipitated from splenocytes by antibodies recognising DEC205 (FG9), CD83 (GE8) and CD11c (8F2). (B) Autoradiographs of proteins immunoprecipitated from lysates of adherent spleen cells labelled with 35S methionine in the presence or absence of LPS. Antibodies used were an isotype-matched control antibody, CC58, the DEC205 and CD11c antibodies and KUL01 (see main text). (A & B) Arrowheads point to specifically precipitated bands.

Methods: (A) Splenocytes were prepared by centrifugation of disaggregated spleen cells onto a Histopaque 1119 cushion, then lysed in 20mM TrisHCl, 10mM EDTA, 100mM NaCl, 0.5% NP40, pH 7.4 containing a cocktail of protease inhibitors (Roche). After preclearing with control agarose resin, immunoprecipitation was carried out with antibodies covalently linked to agarose resin (Pierce Co-IP kit).

(B) Spleen cells released by enzymatic digestion and sedimentation onto Histopaque 1119 (see main text) were incubated at 41°C in methionine-free RPMI 1640 medium containing 10%FCS in plastic culture flasks at 108cells/cm2 for two hours. After washing away non-adherent cells, remaining cells were cultured for 16 hours with 3 mls medium containing 3.7MBq 35S-methionine (Hartman) per 25cm2 flask, with or without 100 ng/ml LPS. Lysates prepared from adherent cells, re-mixed with detached cells recovered by centrifugation, were precleared with 1% v/v protein G sepharose for 16 hrs. Antibody was added at 5 µg/ml and incubated for 30 minutes, then for 60 minutes with 0.75% v/v protein G sepharose.

SDS PAGE were carried out using standard methods after elution of proteins from beads in reducing conditions.

References:

1. http://www.cbs.dtu.dk/services/NetNGlyc/
2. Guo M et al. (2000) Human Immunology 61, 729-738. (figure 3).
3. Kato Kato M et al. (2006) International Immunology 18, 857-869. (figure 1E).
