# Supplementary material for: Expression of Chicken DEC205 Reflects the Unique Structure and Function of the Avian Immune System
Source: PLoS One. 2013 Jan 9;8(1):e51799. doi: 10.1371/journal.pone.0051799 (PMC3541370; doi:10.1371/journal.pone.0051799)
Supplement: Figure S6 — GE8 monoclonal antibody recognises the chicken CD83 gene product expressed on transfected COS cells. (PDF) [file pone.0051799.s006.pdf]

Supplementary figure S6

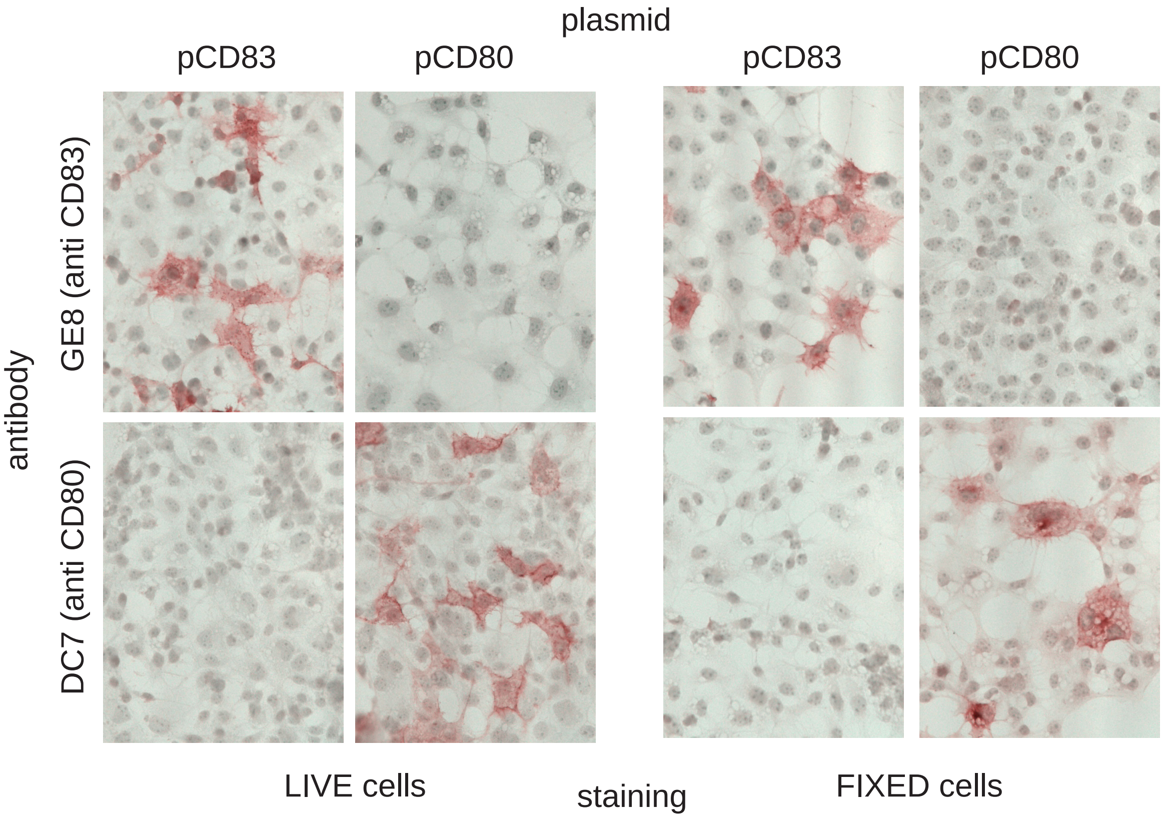

Supplementary figure S6. GE8 monoclonal antibody recognises the chicken CD83 gene product expressed on transfected COS cells.

COS-7 cells transfected with plasmid expression vector containing the full length chicken CD83 coding sequence (pCD83), or a control plasmid (pCD80), were stained, either live or after fixation, with GE8 anti-CD83 monoclonal antibody or the control monoclonal antibody DC7, that recognises the expression product of pCD80. The GE8 antibody stained cells transfected with pCD83, but not cells transfected with control plasmid. The control antibody did not stain the pCD83 transfected cells, but did stain the control transfected cells, demonstrating that they had been successfully transfected. These data show that the anti chicken CD83 monoclonal antibody GE8 does recognise the intact chicken CD83 molecule in its native form on the surface of transfected cells.

Methods:

The pCD83 plasmid was constructed by insertion of the full length coding sequence of chicken CD83 (1), amplified by PCR from a library of cDNA from the macrophage cell line HD11, into the expression vector pCDNA 3.1. After verification by sequencing, the plasmid was transfected into COS-7 cells using Lipofectamine LTX and Plus reagent, as described by the manufacturer (Life Technologies) in 9cm^2 slide flasks (Nunc). At 72 hours after transfection, cells were washed with PBS and incubated with 5 µg/ml antibody in PBS containing 1% BSA and 0.1 % sodium azide on ice for one hour, either before or after fixing in 1:1 acetone/methanol. This was followed by washing in PBS, incubation with polyclonal rabbit anti mouse Ig HRP conjugate (Dako) and detection of HRP using ACE substrate (Calbiochem). Control plasmid pCD80 and control antibody DC7 were provided by JRY (unpublished data).

References:

1. Hansell C et al. (2007) J. Immunol. 179, 5117-5125.
