# Supplementary material for: Expression of Chicken DEC205 Reflects the Unique Structure and Function of the Avian Immune System
Source: PLoS One. 2013 Jan 9;8(1):e51799. doi: 10.1371/journal.pone.0051799 (PMC3541370; doi:10.1371/journal.pone.0051799)
Supplement: Figure S7 — DEC205 expression in non-lymphoid tissues. (PDF) [file pone.0051799.s007.pdf]

Supplementary figure S7

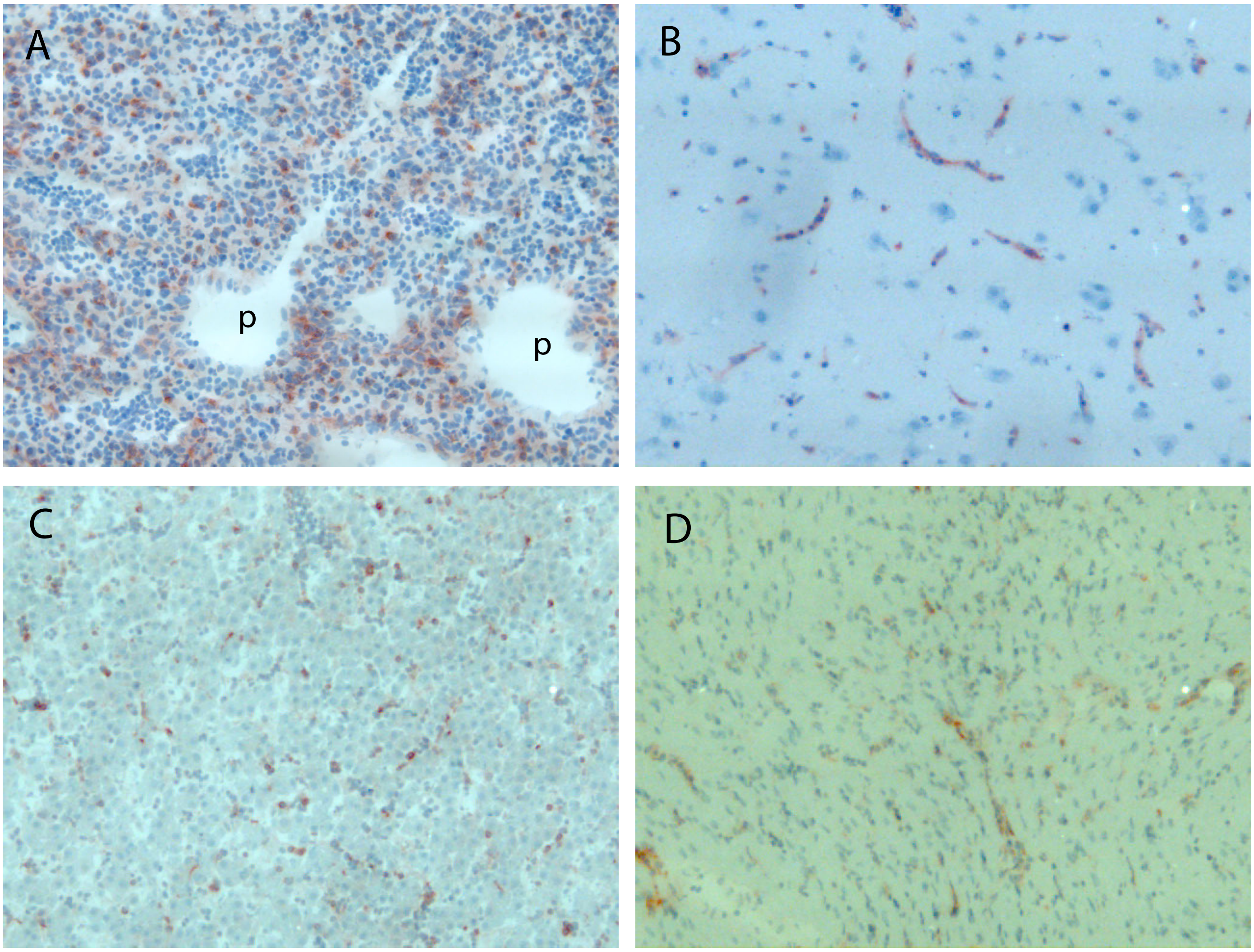

Supplementary figure S7. DEC205 expression in non-lymphoid tissues

A) Lung showing immunoperoxidase staining of scattered cells in the lung parenchyma surrounding parabronchi (p).

B) Apparent staining of the cerebrovascular endothelia of the cerebral cortex

C) Staining of scattered cells in the kidney

D) Apparent staining of vascular endothelia and other cells in cardiac muscle
