# Supplementary material for: Expression of Chicken DEC205 Reflects the Unique Structure and Function of the Avian Immune System
Source: PLoS One. 2013 Jan 9;8(1):e51799. doi: 10.1371/journal.pone.0051799 (PMC3541370; doi:10.1371/journal.pone.0051799)
Supplement: Table S1 — Antibodies. (PDF) [file pone.0051799.s008.pdf]

Supplementary table S1. Antibodies

Ab/Cat No.	Antigen	Use	Class	Conjugate	Supplier
AV29	CD4	IHC	IgG2b	-	IAH
AV14	CD8α	IHC	IgG2b	-	IAH
AV20	Bu-1	IHC	IgG1	-	SB
KUL01	macrophage	IHC/CFM	IgG1	-	SB
2G11	MHC class II	CFM	IgG1	-	J. Kaufman [1]
FG9	DEC205	IHC/CFM	IgG1	-	IAH
AD6	DEC205	CFM	IgG2b	-	IAH
GE8	CD83	IHC/CFM	IgG2a	-	IAH
2040-01	hu IgG	ELISA	poly (goat)	-	SB
2040-05	hu IgG	ELISA	poly (goat)	HRP	SB
1030-05	mo IgG	ELISA	poly (goat)	HRP	SB
A21121	mo IgG1	CFM	poly (goat)	A-488	InVitrogen
A21124	mo IgG1	CFM	poly (goat)	A-568	InVitrogen
A21134	mo IgG2a	CFM	poly (goat)	A-568	InVitrogen
A21141	mo IgG2b	CFM	poly (goat)	A-488	InVitrogen
8210-02	CD4	FC	IgG1	FITC	SB
8280-02	CD8β	FC	IgG2a	FITC	SB
8395-02	Bu-1	FC	IgG1	FITC	SB
8420-02	KUL01	FC	IgG1	FITC	SB
8230-09	TCR1	FC	IgG1	R-PE	SB
8F2	CD11c [2]	FC	IgG2a	AF-488	B. Kaspers [3]
A21236	moIgG (H+L)	FC	Poly(goat)	AF-647	InVitrogen

Abbreviations: IHC Immunohistochemistry, CFM confocal Microscopy, FC Flow cytometry; HRP horse radish peroxidase, R-PE rhodamine-phycoerythrin, AF Alexa Fluor (InVitrogen); IAH Institute for Animal Health (Compton, UK), SB Southern Biotechnology Associates (Birmingham, AL). Footnotes: (1) A generous gift from J. Kaufman, University of Cambridge, UK. (Kaufman et al. 1990, *J. Immunol* 133, 2258-2272). (2) Putative anti-CD11c. (3) A generous gift from Prof. Bernd Kaspers, Institut für Tierphysiologie,University of Munich, Germany.
