# Supplementary material for: Expression of Chicken DEC205 Reflects the Unique Structure and Function of the Avian Immune System
Source: PLoS One. 2013 Jan 9;8(1):e51799. doi: 10.1371/journal.pone.0051799 (PMC3541370; doi:10.1371/journal.pone.0051799)
Supplement: Table S2 — Primers used for determining the chicken DEC205 sequence. (PDF) [file pone.0051799.s009.pdf]

Supplementary table S2. Primers used for determining the chicken DEC205 sequence

Name	sequence	Orientation	Use
DEC205-1-For	5’-GCCAGCTCTCAACATTGCTAC	Forward	cDNA PCR
DEC205-1-Rev	5’-ACCCACCCACACTTCTTCTC	Reverse	cDNA PCR
Cons-C-For1	5’-TTCACCATCAGGCACGACAC	Forward	cDNA PCR
Cons-C-Rev1	5’-TCCCAACCCTCATCCAATGAGC	Reverse	cDNA PCR
Cons-B-For1	5’-GCCTTGCCTGCTACAAGTTC	Forward	cDNA PCR
Cons-B-Rev1	5’-GCAGACCCATTCAAGTTTAGCC	Reverse	cDNA PCR
Cons-B-For2	5’-CATCCTTGCTTCCTCAAATGCC	Forward	cDNA PCR
Cons-B-Rev2	5’-ACACGCACTCTTCGCTTGTC	Reverse	cDNA PCR
BR2-For	5’-GACAAGCGAAGAGTGCGTGT	Forward	cDNA PCR
AF2-Rev	5’-GGCTCGGCTCACTTGCTTTT	Reverse	cDNA PCR
AF1-Rev	5’GTCAGAAGGGCTGCTGAAGGAT	Reverse	cDNA PCR
Cons-A-For2	5’-AAAAGCAAGTGAGCCGAGCC	Forward	cDNA PCR
Cons-A-For1	5’-ATCCTTCAGCAGCCCTTCTGAC	Forward	cDNA PCR
Cons-A-Rev2	5’-TGAAGTTCGTGCTGAATCTCCC	Reverse	cDNA PCR
Cons-A-Rev1	5’-ACAGCTCCAAAACCCTTGTACC	Reverse	cDNA PCR
DEC205-BAC1R	5’-TTAACTTGTATGCACTTG	Reverse	BAC walking
DEC205-BAC1F	5’-CAAGTGCATACAAGTTAA	Forward	BAC walking
DEC205-BAC2R	5’-CTGGGACACCCATTTCCA	Reverse	BAC walking
DEC205-BACR3	5’-AAGCAACTCTAACAACCCGT	Reverse	BAC walking
DEC205-BACR4	5’-TTCTACCGTGCCCTACGCCTC	Reverse	BAC walking
DEC205-BACR5	5’-CTCTTCGCTCTTCTTCCCGTC	Reverse	BAC walking
DEC205-BACR7	5’-TCCCGCCGGCCGCCCCAT	Reverse	BAC walking
DEC205F-NheI	5’-CTCCCAAAGGCTAGCGATCCCTGTTCC	Forward	cDNA PCR
DEC205R-BglII	5’-TGTCTGGAGATCTGCAGCTCCTA	Reverse	cDNA PCR
CD83R-BglII	5’-AGCATGAAAAGATCTGTCTTGTAT	Reverse	cDNA PCR
CD83F-NheI	5’-TGATTTGAGCTAGCAATACCCATG	Forward	cDNA PCR
