# Supplementary material for: Expression of Chicken DEC205 Reflects the Unique Structure and Function of the Avian Immune System
Source: PLoS One. 2013 Jan 9;8(1):e51799. doi: 10.1371/journal.pone.0051799 (PMC3541370; doi:10.1371/journal.pone.0051799)
Supplement: Table S3 — Quantitative reverse transcription-PCR primer and probe sequences. (PDF) [file pone.0051799.s010.pdf]

Supplementary table S3. Quantitative reverse transcription-PCR primer and probe sequences

**Gene**	**Type**	**Sequence**	**Exons**
DEC205	Forward	5'-GGAATGAATAAGAGGAGTCCAGATTC-3'	14
DEC205	Reverse	5'-CAAGACAAGACTGGTTACGGGTTTA-3'	14,15
DEC205	Probe	5'(FAM)-CGTCACTCCATTGCCAGGTTCCCA-(TAMRA)-3'	14
CD83	Forward	5'-CACCCTGTGCAATGTTTGGA-3'	1,2
CD83	Reverse	5'-CAAAGCATGTCACAGCAACATCT-3'	2
CD83	Probe	5'-(FAM)-ACAGCCACAGCAGCTCCATTGATCAAG-(TAMRA)-3'	2
28S	Forward	5'-GGCGAAGCCAGAGGAAACT-3'	
28S	Reverse	5'-GACGACCGATTTGCACGTC-3'	
28S	Probe	5'-(Yakima yellow)-AGGACCGCTACGGACCTCCACCA-(TAMRA)-3'	
